# Kikuchi-Fujimoto disease associated with community acquired pneumonia showing intrathoratic lymphadenopathy without cervical lesions

**DOI:** 10.1186/s40064-015-1500-y

**Published:** 2015-11-11

**Authors:** Nobuhito Naito, Tsutomu Shinohara, Hisanori Machida, Hiroyuki Hino, Keishi Naruse, Fumitaka Ogushi

**Affiliations:** Division of Pulmonary Medicine, National Hospital Organization National Kochi Hospital, 1-2-25 Asakuranishimachi, Kochi, 780-8077 Japan; Department of Clinical Investigation, National Hospital Organization National Kochi Hospital, 1-2-25 Asakuranishimachi, Kochi, 780-8077 Japan; Division of Thoracic Surgery, National Hospital Organization National Kochi Hospital, 1-2-25 Asakuranishimachi, Kochi, 780-8077 Japan; Division of Pathology, National Hospital Organization National Kochi Hospital, 1-2-25 Asakuranishimachi, Kochi, 780-8077 Japan

**Keywords:** Kikuchi-Fujimoto disease, Community acquired pneumonia, Intrathoratic lymphadenopathy

## Abstract

**Introduction:**

Kikuchi-Fujimoto disease (KFD), or histiocytic necrotizing lymphadenitis, is a rare entity of unknown etiology in young adults that is typically characterized by cervical lymphadenopathy and persistent fever. The pathogenesis of KFD has been suggested to be an abnormal immune response, and infections or autoimmune diseases are considered to be involved in KFD. However, KFD associated with community acquired pneumonia (CAP) has not been reported.

**Case description:**

A 35-year-old male was admitted due to high fever, diffuse air-space consolidation in the right lung with ipsilateral pleural effusion and massive mediastinal and hilar lymphadenopathy without cervical lesions. On clinical suspicion of malignant lymphoma complicated with pneumonia, we performed a video-assisted thoracoscopic lymph node biopsy, and the diagnosis of KFD was established. Complete cure of the intrathoratic lesions was observed by administration of β-lactam antibiotics alone without steroid therapy.

**Discussion and evaluation:**

Previous large case series have identified no pathogenic relationship between KFD and pneumonia. The hilar adenopathy could have caused airway compression leading to pneumonia.

**Conclusions:**

KFD should be considered in the differential diagnosis of massive mediastinal and hilar lymphadenopathy, even when there are no superficial lesions. In addition, we need to bear in mind that unexpected disorders occasionally coexist with common diseases.

## Background

Kikuchi-Fujimoto disease (KFD) is a clinicopathological entity typically characterized by cervical lymphadenopathy, persistent fever, and histiocytic necrotizing lymphadenitis of unknown etiology in young adults. The histopathologic changes can be classified into three histologic types; proliferative, necrotizing, and xanthomatous types (Kuo 1995). The clinical course of KFD is relatively benign and typically self-limiting, although corticosteroid therapy has been used for some patients with complicated KFD (Jang et al. 2000; Yoshioka et al. 2010). Generally, KFD localizes in the cervical region, and systemic lymph nodes involvement is unusual (Dumas et al. 2014; Nakamura et al. 2009). Especially, deep lesions without cervical lymphadenopathy are extremely rare (Yoshida et al. 2011). The pathogenesis of KFD has been suggested to be an abnormal immune response, and infections (e.g., Epstein-Barr virus, parainfluenza virus, human herpes virus, human immunodeficiency virus, Toxoplasma and Yersinia enterocolitica) or autoimmune diseases (e.g., systemic lupus erythematosus) are considered to be involved in KFD (Dumas et al. 2014). However, KFD associated with community acquired pneumonia (CAP) has not been reported.

## Case description

**Fig. 1:**
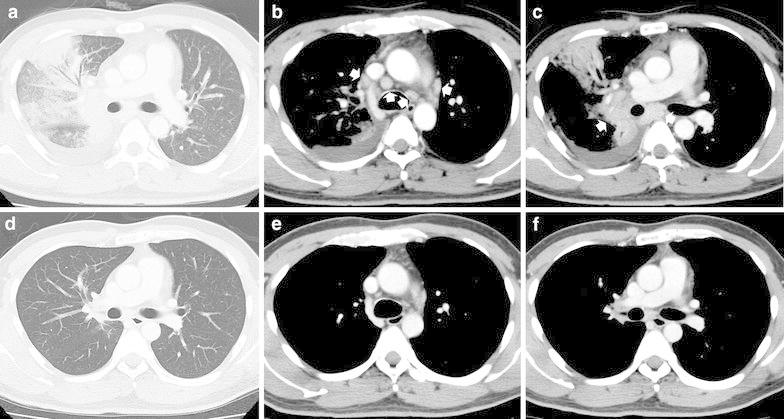
Chest enhanced CT images (**a**, **d** lung parenchyma window; **b**, **c**, **e**, **f** mediastinal window). **a**–**c** Diffuse air-space consolidation in the right lung with ipsilateral pleural effusion and massive mediastinal and hilar lymphadenopathy (*arrows*) at admission. **d**–**f** Disappearance of consolidation, pleural effusion and lymphadenopathy after the treatment

**Fig. 2:**
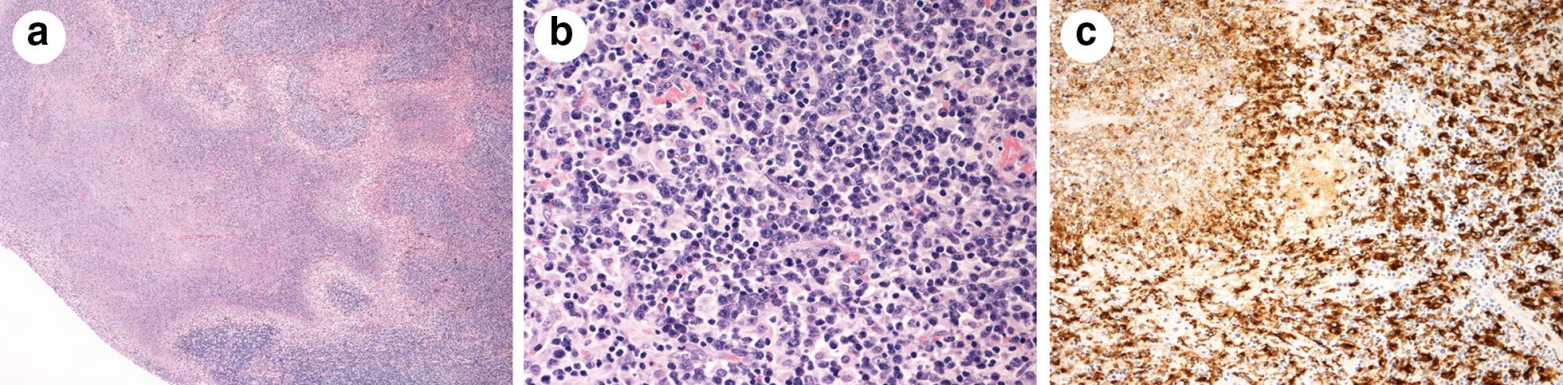
Pathological findings of mediastinal lymph nodes (**a**, **b** hematoxylin and eosin staining: **c** immunohistochemical staining). **a** Irregular necrotic areas consisted of debris and intense karyorrhexis. **b** Prominent histiocytic cells admixed with plasma cells, large lymphocytes and immunoblasts around necrotic areas without neutrophils. **c** Positive stain for CD68 on histiocytic cells

A 35-year-old male with complaints of high fever and malaise for 3 days visited a nearby clinic, where antibiotic therapy (ceftriaxone 1 g/day) was started based on leukocytosis (10,000/µl) and elevated serum C-reactive protein (CRP) level (7.39 mg/dl). Two days later, he was referred to our hospital for further examination. Physical examination indicated reduced respiratory sounds in the left lung. Hepatosplenomegaly and superficial lymphadenopathy were not observed. Chest X-ray and enhanced computed tomography (CT) showed diffuse air-space consolidation in the right lung with ipsilateral pleural effusion and massive mediastinal and hilar lymphadenopathy (Fig. 1a–c). Elevated serum levels of AST (206 U/L), ALT (286 U/L), LDH (446 U/l) and soluble interleukin-2 receptor (sIL-2R) (2550 U/ml), but not angiotensin converting enzyme (7 IU/l), were observed. A thoracentesis revealed neutrophilic effusion with negative cytology. Sputum, blood, and pleural effusion cultures of bacteria using conventional methods were negative, probably due to antibiotic therapy started before collection of the specimens. On clinical suspicion of malignant lymphoma (ML) complicated with pneumonia, we performed a video-assisted thoracoscopic lymph node biopsy while continuing administration of β-lactam antibiotics (tazobactam/piperacillin: TAZ/PIPC 13.5 g/day). Histological examinations of the biopsy specimens revealed irregular necrotic areas that consisted of debris and intense karyorrhexis. Prominent histiocytic cells admixed with plasma cells, large lymphocytes and immunoblasts were observed around these areas without neutrophils. Lymphoma cells were not detected. Immunohistochemical studies showed histiocytic cells were strongly positive for CD68 (Fig. 2a–c). These histological findings were compatible with the necrotizing type of KFD. When the final pathological diagnosis was established, the clinical course had already turned favorable. Therefore, we continued administration of TAZ/PIPC alone for a total period of 10 days without steroid therapy. Two weeks after the admission, leukocytosis and serum levels of CRP and LDH were reduced to a normal range. Elevated liver enzymes, observed in 24 % of patients with KFD (Dumas et al. 2014), disappeared. sIL-2R, which was very high for KFD (Nakamura et al. 2009), also normalized after 1 month. Follow-up chest CT showed no consolidation, pleural effusion and lymphadenopathy (Fig. 1d–f). Antibody titers for Epstein-Barr virus using a fluorescent antibody method and the particle agglutination titer for *Mycoplasma pneumoniae* did not rise during the treatment. At 2 years’ follow-up, no evidence of recurrence was detected.

## Discussion

In the present case, physical examination and enhanced CT showed no lymphadenopathies except for intrathoratic lesions. Although several cases of KFD with lymphadenopathy in atypical location(s) have been reported (González-Ballester et al. 2010; Aneja et al. 2014), the presence of cervical lymphadenopathy was a common and distinctive feature of KFD excluding a few cases (Dumas et al. 2014; Nakamura et al. 2009). Previously, Yoshida et al. reported mediastinal lymphadenopathy without cervical adenopathy in middle-aged men diagnosed with KFD by mediastinal lymph node biopsy (Yoshida et al. 2011). However, the patient had no respiratory symptoms and concomitant diseases. On the other side, a few cases of KFD, diagnosed by cervical lymph node biopsy, presented with interstitial pneumonia (Garcia-Zamalloa et al. 2010) or cryptogenic organizing pneumonia (COP) (Hua and Zhu 2010), and were successfully treated with oral prednisone (1 mg/kg/day). However, to the best of our knowledge, this manuscript is the first case report of KFD associated with CAP, which is also extremely rare in the location of lymphadenopathy.

The complete disappearance of diffuse consolidations by β-lactam antibiotic and parapneumonic effusion with neutrophilic predominance suggested that the etiology of CAP in this case was bacterial infection. In addition, acute illness, high fever, pleural effusion and the unilaterality of consolidation observed in this case are atypical findings for COP (Hua and Zhu 2010). Although the localization of histiocytic necrotizing lymphadenitis in the regional lymph nodes of the lung was observed, previous large case series have identified no pathogenic relationship between KFD and pneumonia (Dumas et al. 2014; Nakamura et al. 2009). The hilar adenopathy could have caused airway compression leading to pneumonia, but we did not confirm the airway stenosis by bronchoscopic examination.

The diagnostic difficulty was differentiating this case from ML. Although 18-fluorodeoxyglucose positron emission/computed tomography (FDG PET/CT) tends to be done to distinguish benign from malignant lesions, conventional or dual phase FDG PET/CT does not necessarily distinguish sarcoidosis and tuberculosis from ML in the mediastinal region (Kumar et al. 2011; Maturu et al. 2014). Therefore, we performed a video-assisted thoracoscopic lymph node biopsy without FDG PET/CT examination. In addition, it was recently reported that FDG PET/CT findings of KFD are indistinguishable from those of ML which has no extranodal involvement (Kim et al. 2014). Endobronchial ultrasound with transbronchial needle aspiration is a promising alternative method for diagnosis of mediastinal and hilar lymphadenopathy due to ML, nonlymphoma malignancy and sarcoidosis (Boujaoude et al. 2012). However, the tissue samples obtained by this method are too small to diagnose KFD.

## Conclusion

KFD should be considered in the differential diagnosis of massive mediastinal and hilar lymphadenopathy, even when there are no superficial lesions. More molecular biological research is needed to obtain insight into the essential pathogenic factors involved in KFD in order to develop a simple diagnostic method as a definitive diagnosis of KFD in the mediastinal and hilar regions is currently only possible by examining surgical biopsy tissue. In addition, we need to bear in mind that unexpected disorders occasionally coexist with common diseases.

## Consent

Written informed consent was obtained from the patient for publication of this case report and any accompanying images. A copy of the written consent is available for review by the Editor-in-Chief of this journal.

## Abbreviations

KFD: Kikuchi-Fujimoto disease
CAP: community acquired pneumonia
CRP: C-reactive protein
CT: computed tomography
sIL-2R: soluble interleukin-2 receptor
ML: malignant lymphoma
TAZ/PIPC: tazobactam/piperacillin
COP: cryptogenic organizing pneumonia
FDG PET/CT: 18-fluorodeoxyglucose positron emission/computed tomography
